# Mannose-Binding Lectin (*MBL*) gene polymorphisms in susceptibility to pulmonary tuberculosis among the Lur population of Lorestan Province of Iran

**DOI:** 10.1016/j.gdata.2017.05.005

**Published:** 2017-05-04

**Authors:** Ali Amiri, Toomaj Sabooteh, Farhad Shahsavar, Khatereh Anbari, Flora Pouremadi

**Affiliations:** a Department of Internal Medicine, Lorestan University of Medical Sciences, Khorramabad, Iran; b Faculty of Medicine, Lorestan University of Medical Sciences, Khorramabad, Iran; c Department of Immunology, Lorestan University of Medical Sciences, Khorramabad, Iran; d Social Determinants of Health Research Center, Lorestan University of Medical Sciences, Khorramabad, Iran; e Student Research Committee, Lorestan University of Medical Sciences, Khorramabad, Iran

**Keywords:** MBL, Pulmonary tuberculosis, Lur population

## Abstract

**Objective:**

Tuberculosis (TB) is caused by infection of Mycobacterium tuberculosis. Host genetic variability is an important determinant of the risk of developing TB in humans. Although the association between *MBL* polymorphisms and TB has been studied in various populations, the results are controversial. The aim of this study was to investigate mannose-binding lectin (*MBL*) gene polymorphisms with susceptibility to pulmonary tuberculosis (PTB) in a Lur population of Iran.

**Methods:**

In this case-control study, four functional *MBL* gene polymorphisms (*HL*, *XY*, *PQ* and *AB*) were genotyped by using PCR Single Strand Conformation Polymorphism (SSCP) technique in a Lur population living in Lorestan Province, consisting of 100 patients with pulmonary tuberculosis (PTB) age and sex matched 100 healthy controls (HCs). Association analyses were performed with the SPSS 21 statistical software.

**Results:**

We found that *MBL* (*HH*) genotype polymorphism significantly was associated with increased susceptibility to TB (35% in patients vs. 22% in controls, P = 0.0417, OR = 1.909, %95 CI = 1.020–3.573). Additionally, *H* allele showed a significant association with increased risk of TB (56.5% in patients vs. 46% in controls, P = 0.0357, OR = 1.525, %95 CI = 1.028–2.262). Also, the distribution of *L* allele in patients was significantly lower frequency in TB patients compared to controls (43.5% vs. 54%, P = 0.0357, OR = 0.656, %95 CI = 0.442–0.973). However, the allelic and genotypic frequencies of *AB*, *XY* and *PQ* polymorphisms were not significantly different between the patients and the controls. We couldn't detect any significant differences between haplotypes among TB patients and healthy controls.

**Conclusions:**

Our findings demonstrated that *HH* genotype and *H* allele may increase the susceptibility to pulmonary TB in the Lur population of Iran, although *L* allele may decrease the susceptibility to pulmonary TB in this population. We suggest that it is necessary to further more studies with larger sample size and other ethnic population.

## 1 Introduction

Tuberculosis (TB) is caused by the infection of Mycobacterium tuberculosis (MTB) and remains the first leading cause of global death from infectious diseases [1]. Each year > 9 million novel cases are infected by PTB and > 1.7 million succumb to PTB annually [2]. Tuberculosis (TB) is responsible for an estimated 1.5 million deaths in 2014 [3]. Approximately 10% of patients who are infected with MTB are known to progress to clinical disease [4], [5]. MTB presumably infects a third of the world's population [6]. Additionally, the course and duration of disease vary in different individuals [7]. This suggests that individual differences may act upon the susceptibility to tuberculosis and that contact with this microorganism does not always result in infection. These differences may be due to host factors and genetic sensitivity of different individuals to this disease [8], [9]. From the anthropological point of view, susceptibility to infectious diseases can be associated with genetic diversities of polymorphic genes like human leukocyte antigen (*HLA*) [10]. The identification of factors which increase disease susceptibility has potential to inform control strategies.

The host-pathogen interactions and environmental factors may contribute to TB. Furthermore, Host genetic variability is an important determinant of the risk of developing TB in humans [11], [12]. Many studies have reported the association between TB and genetic polymorphisms related to human innate immunity [7]. Unraveling the genetic factor contributing to the pathogenesis of TB may lead to improved treatment and prevention of this disease [13]. One of which is *KIR3DS1* gene and it combines with *HLA-B Bw4* and *Ile80* ligand [14], [15]. Also, other studies showed Toll like Receptors (TLRs) [16], [17], [18], [19], [20], Interlukin-10 (IL-10) [21], Tumor Necrosis Factor (TNF) [22] and Vitamin D Receptor (VDR) has roles in the susceptibility or resistance to TB [23], [24].

Mannose-Binding Lectin (MBL) is a member of the collectin family that recognizes pathogens by its carbohydrate-recognition domains [25]. MBL is an acute-phase serum protein, which is synthesized in the liver and circulates as oligomers complexed with MBL-associated serine proteases (MASPs) [26]. MASPs bind to the sugar moieties on the surface of a pathogen, and then are activated to initiate the lectin pathway of complement activation, resulting in opsonization and phagocytosis or lysis of pathogens. In addition, MBL can regulate inflammatory responses and immune activation [26]. Case control studies have previously reported independent associations between *MBL* polymorphisms and susceptibility to active TB.

*MBL2* gene (10q11.2-q21) encodes for the mannose binding lectin and presents several polymorphisms, four of which are known for their functional effect [27]. Four single-nucleotide polymorphisms (SNPs) in exon 1 (codon 54 *AB* (rs1800450)), and in the promoter and 5′-untranslated regions (nt − 550 *HL* (rs11003125), nt − 221 *XY* (rs7096206), and nt + 4 *PQ* (rs7095891)) of the *MBL2* gene are associated with serum levels and/or functions of MBL. Many studies showed these genetic variations are associated with a wide variety of diseases, including respiratory tract infections [28], [29], [30], [31]. So, *MBL* polymorphisms may contribute to pulmonary tuberculosis susceptibility.

Previous studies have not reached a consensus regarding the association between the *MBL* gene polymorphisms and the susceptibility to PTB. However, in previously reported studies *MBL2* polymorphisms have conflicting results showing protection from or susceptibility to TB [7]. For example, low levels of MBL were reported to protect against tuberculosis [13], [32]. Other investigators instead claim that protection against the disease is associated with high levels of MBL [33], [34], [35], [36]. In the Chinese Han population, no convincing evidence of association between *MBL2* sequence variants and TB was observed [37], [38], [39], [40], [41]. Shi et al. reported that variants at *AB* were associated with increased susceptibility to TB in Chinese origin [38]. Additionally, Chen et al. found that variants in *YX* were associated with increased susceptibility to TB among Chinese [40], [41]. However, most recently Wu et al. reported that variants in *HL*, but not *YX*, *PQ* or *AB*, were associated with decreased susceptibility to TB among Chinese [37]. Many studies have examined the potential contribution made by *MBL* polymorphisms to PTB susceptibility, but the findings have been contradictory.

Conflicting results are not unexpected in association studies for several reasons, including small sample size, marginal statistical significance, detection of genotypes, or ethnic heterogeneity. Therefore, here we conducted a genetic association case-control study in a Lur population of Lorestan origin, to define the association between the *MBL* polymorphisms and TB.

## 2 Materials and methods

### 2.1 Patients and controls

The study was approved by the Ethical Committee of Lorestan University of Medical Sciences and Informed consent was taken from all subjects before blood sampling and questionnaire investigation. All subjects agreed to take part in the study. The patients included in this study were newly diagnosed PTB patients registered in the health center of Khorramabad city of Lorestan Province from January 2016 to January 2017. Cases were 100 unrelated Lur individuals selected with newly diagnosed pulmonary tuberculosis, positive sputum smear and/or culture and significant symptoms of typical PTB, chest radiography consistent with active disease. Then, patients with any autoimmune disease, chronic renal failure and any chronic inflammatory disease were excluded. The control group composed of healthy individuals who matched in age and gender. Controls were 100 unrelated Iranian individuals of the same race and geographic region. None of the controls showed any clinical manifestations of PTB at the time of blood sample collection, and no evidence of prior PTB noted on chest radiography. The healthy control group composed of healthy individuals who matched in age and gender with the patient group. Additionally, all study subjects had parents of the same race.

### 2.2 Determination of sample size

The sample size was determined by the following factors: the known prevalence *MBL* genetic polymorphisms in the Iranian population, α and β errors. We used a value of at least 10% for the prevalence of polymorphisms, an α error of 0.05, a β error of 0.2, a 10% expected difference, and match ratio of 1:1. The minimum sample size was estimated to be 100 for the PTB group and 100 for the HC group.

### 2.3 Genotyping

A case-control study was conducted in 100 pulmonary tuberculosis (PTB) patients and 100 healthy controls (HCs). Blood samples were collected from all subjects. We extracted genomic DNA from 5 ml Venous blood samples using the QIAmp kit (Qiagen, Germany) according to the manufacturer's protocol. Polymerase chain reaction (PCR) and single-strand conformation polymorphism (SSCP) technique were used to study the frequencies of *MBL* gene polymorphisms, that previously suggested by Wu et al. [37] and Chen et al. [40]. *MBL* gene, the *AB*, *HL*, *PQ* and *XY* polymorphisms were detected in patients and controls using their genomic DNA. PCR amplifications were performed using purified DNA Mastercycler (BioRad, USA) in 25 μl reaction volumes. Thermocycling parameters were as follows: 94 °C for 4 min and 35 cycles of 94 °C for 30 s, 59 °C for 60 s and 72 °C for 45 s, with a final extension at 72 °C for 5 min. PCR amplification products were analyzed on a polyacrylamide gel to detect alterations in PCR-amplified products. The primer sequences were previously reported [27], [39], [42] and listed in Table 1. To ensure the validity of result, we also did the DNA sequencing on the 5% representative isolates to check the PCR.

**Table 1:** Primer sequences for genotyping of *MBL* polymorphisms.

MBL polymorphisms	Sequences of the primers
MBL-AB (+ 230) (rs1800450)	F: 5′-AGTCGACCCAGATTGTAGGACAGAG-3′
	R: 5′-AGGATCCAGGCAGTTTCCTCTGGAAGG-3′
MBL-HL (− 550) (rs11003125)	F: 5′-GCTTACCCAGGCAAGCCTGTG-3′
	R: 5′-ACTTACCCAGGCAAGCCTGTC-3′
MBL-PQ (+ 4) (rs7095891)	F: 5′-CTCAGTTAATGAACACATATTTACCG-3′
	R: 5′-CTCAGTTAATGAACACATATTTACCA-3′
MBL-XY (− 221) (rs7096206)	F: 5′-GGTCCCATTTGTTCTCACTCCCACC-3′
	R: 5′-GAAAGCATGTTTATAGTCTTCCAGC-3′

### 2.4 Statistical analysis

The genotypic and allelic frequencies of *AB*, *HL*, *PQ* and *XY* polymorphisms were ascertained by direct counting in the PTB patients and control group. Departure from Hardy-Weinberg Equilibrium (HWE) of All polymorphisms frequency was assessed by an exact test in both patient and control groups. Data were managed and analyzed using SPSS 21 program. We calculated differences in the genotypic and allelic frequencies of *AB*, *HL*, *PQ* and *XY* polymorphisms between the cases group and control group by Chi-square test and Fisher's exact test [43]. All of the P-values presented in this study are two-sided, and P ≤ 0.05 was used as the threshold of statistical significance. Odds ratios (OR) and 95% confidence intervals (CIs) were calculated to quantify the degree of association between the polymorphisms and tuberculosis [44].

## 3 Result

A total of 100 cases with smear-positive PTB and 100 healthy controls were recruited to the study. The mean age of cases was (28.83 ± 6.12, range 21–46 years) and the mean age of controls was (26.18 ± 4.35, range 19–53 years). 46 individuals of cases were males and 54 individuals were females, and 58 individuals of controls were males and 42 individuals were females.

The genotype distribution of *MBL* polymorphisms in all subjects did not deviate from the Hardy-Weinberg equilibrium (P > 0.05). The results of the association between the *MBL AB* (+ 230) (rs1800450), *HL* (− 550) (rs11003125), *PQ* (+ 4) (rs7095891) and *XY* (− 221) (rs7096206) genotypic and allelic frequencies and the risk of PTB were listed in Table 2, Table 3.

**Table 2:** Distribution of MBL genotypes in TB patients group and healthy controls group.

MBL polymorphisms	Genotypes	% of TB patients group	% of healthy controls group	OR	P value
MBL-AB	AA	69	72	0.866	0.6418
	AB	29	27	1.104	0.7528
	BB	2	1	2.020	0.5607
MBL-HL	HH	35⁎	22	1.909	0.0417⁎
	HL	43	48	0.817	0.4777
	LL	22	30	0.658	0.1972
MBL-PQ	PP	76	69	1.423	0.2676
	PQ	21	26	0.757	0.4044
	QQ	3	5	0.588	0.4705
MBL-XY	XX	5	7	0.699	0.5515
	XY	36	29	1.377	0.2906
	YY	59	64	0.809	0.4675

⁎ Significant difference (P < 0.05), n = 100.

**Table 3:** Distribution of MBL alleles in TB patients group and healthy controls group.

MBL polymorphisms	Alleles	% allele frequency in TB patients group	% allele frequency in Healthy controls group	OR	P value
MBL-AB	A	83.5	85.5	0.858	0.5805
	B	16.5	14.5	1.165	0.5805
MBL-HL	H	56.5⁎	46	1.525	0.0357⁎
	L	43.5⁎	54	0.656	0.0357⁎
MBL-PQ	P	86.5	82	1.407	0.2167
	Q	13.5	18	0.711	0.2167
MBL-XY	X	23	21.5	1.091	0.7184
	Y	77	78.5	0.917	0.7184

⁎ Significant difference (P < 0.05).

In PTB patients, the *HH*, *HL*, *LL* genotype frequencies of the *MBL* gene were 35%, 43% and 22%, while these were 22%, 48% and 30% in healthy individuals respectively. The genotypic frequencies of *MBL AB*, *PQ* and *XY* polymorphisms did not have significant difference between the PTB and the controls. Based on the analysis of loci, we found that MBL (HH) genotype polymorphism significantly was associated with increased susceptibility to TB (35% in patients vs. 22% in controls, P = 0.0417, OR = 1.909, %95 CI = 1.020–3.573). Additionally, *H* allele showed a significant association with increased risk of TB (56.5% in patients vs. 46% in controls, P = 0.0357, OR = 1.525, %95 CI = 1.028–2.262). Also, the distribution of *L* allele in patients was significantly lower frequency in TB patients compared to controls (43.5% vs. 54%, P = 0.0357, OR = 0.656, %95 CI = 0.442–0.973). For MBL-HL (rs11003125) polymorphism, the genotype (*HH*) and *H* allele increased PTB risk by 1.909-fold and 1.525-fold respectively among patients compared with controls. Also, the *L* allele decreased PTB risk by 0.656-fold among patients compared with controls (Table 2). The allelic frequencies of MBL *AB*, *PQ* and *XY* polymorphisms did not have significant difference between the patients and the controls (Table 3). The combination of the seven haplotypes found in patients and controls are shown in Table 4. In the present study we couldn't detect any significant differences between haplotypes among TB patients and healthy controls.

**Table 4:** Frequencies of complete diplotypes in Lur tuberculosis (TB) patients and controls.

MBL2 diplotypes	TB patients (%)	Controls (%)	P values	OR	95% CI
HYPD/HYPA	2	2	1.0000	1	0.138–7.242
HYPD/HYPD	4	2	0.4071	2.041	0.365–11.409
HYPA/LYQC	2	1	0.5607	2.020	0.18–22.647
HYPA/HYPA	2	4	0.4071	0.489	0.087–2.736
LYPB/HYPA	7	5	0.5515	1.430	0.438–4.666
LYPB/LYPB	5	3	0.4705	1.701	0.395–7.321
LYPB/LYQC	3	2	0.6506	1.515	0.247–9.27
LYPB/HYPD	3	2	0.6506	1.515	0.247–9.27
LXPA/HYPA	8	11	0.4694	0.703	0.27–1.83
LXPA/LYQA	9	8	0.7998	1.137	0.42–3.077
LXPA/LXPA	6	7	0.7742	0.848	0.274–2.618
LXPA/LYPB	7	9	0.6022	0.761	0.271–2.13
LXPA/HYPD	3	3	1.0000	1	0.196–5.077
LXPA/LYPA	3	2	0.6506	1.515	0.247–9.27
LXPA/LYQC	3	1	0.3124	3.061	0.313–29.95
LYQA/HYPA	11	12	0.8246	0.906	0.38–2.163
LYQA/LYPB	1	4	0.1742	0.242	0.026–2.208
LYQA/LYPA	3	3	1.0000	1	0.196–5.077
LYQA/LYQA	1	5	0.0973	0.191	0.022–1.673
LYQA/HYPD	0	2	–	–	–
LYQA/LYQC	0	1	–	–	–
LYQC/HYPD	2	1	0.5607	2.020	0.18–22.647
LYPA/HYPA	4	3	0.7004	1.347	0.293–6.18
LYPA/LYPB	3	1	0.3124	3.061	0.313–29.95
LYPA/LYPA	4	3	0.7004	1.347	0.293–6.18
LYPA/LYQC	2	2	1.0000	1	0.138–7.242
LYPA/HYPD	2	1	0.5607	2.020	0.18–22.647
Total	100	100			

No Significant difference between TB patients and controls.

## 4 Discussion

Tuberculosis is an infectious disease caused by Mycobacterium tuberculosis. However, a relatively small proportion of people infected with Mycobacterium tuberculosis will develop TB [45], [46], [47], [48]. Host genetic factors can determine differences in the susceptibility and/or resistance to infections, as well as in the clinical patterns of diseases. The host immune response against MTB is mediated by cellular immunity [11]. Recently, *MBL2* gene polymorphisms have been reported to be associated with the risk of TB. However, the results were inconsistent and inconclusive *MBL* gene may be involved in genetic susceptibility to TB. They are rapidly degraded and exist as lower order oligomers, which have a lower binding capacity to mannose and do not activate complement [49]. MBL has been shown to bind with lipoarabinomannan, one component of Mycobacterium tuberculosis, and promote opsonophagocytosis [50], [51]. Low serum level of the protein may contribute to the susceptibility to TB. In previous studies, there is no research reported that the association between *MBL* gene and PTB susceptibility in the Lur population of Iran. We for the first time studied the correlations between *MBL* gene polymorphisms *AB* (+ 230) (rs1800450), *HL* (− 550) (rs11003125), *PQ* (+ 4) (rs7095891) and *XY* (− 221) (rs7096206) and PTB susceptibility in this population. In our study we showed that *MBL* (*HH*) genotype and the *H* allele are involved in the susceptibility to TB development in a Lur population of Iran. A significant association was found for *L* allele to resistant against the pulmonary tuberculosis. The genotypic and allelic frequencies of *MBL AB*, *XY* and *PQ* polymorphism showed no correlations with PTB susceptibility By contrast, our study did not replicate previous studies of associations between MBL polymorphisms and susceptibility to PTB.

The relation between MBL gene polymorphisms and susceptibility to TB has been studied in different populations. The effect of low MBL levels on TB has been controversial. Some studies reported low MBL levels as associated with protection against the disease, whereas others estimated a relation with increased susceptibility [52]. Denholm et al. suggested that there was no significant association between *MBL* gene polymorphism and pulmonary TB infection [52]. Studies showed that *MBL-AB* polymorphism, affect the serum level as well as configuration and function of MBL more significantly than other [25]. In China, the *MBL-AB* gene polymorphism is more common than the other [39]. Alagarasu et al. [53] and Selvaraj et al. [54], have previously suggested that *BB* genotype may be associated with susceptibility to TB, since they observed a significant increase in the frequency of *BB* genotype in TB patients than controls. Additionally, Capparelli et al. [33], reported higher frequencies of the *BB* genotype among patients (22.3% vs. 3.5%in controls) in an Italian population. Our results are inconsistent with some previous studies, a possible explanation could be their ethnic differences.

On other hand, some studies have shown that MBL deficiency protects against disease caused by M. tuberculosis [39], [52], [55], [56]. *MBL2* variants, either structural alleles (codons 54) in Gambian children [57] and South African adults [58], or full promoter haplotypes responsible for low MBL production, have been shown to be protective against tuberculosis. Søborg et al. [13] demonstrated a significantly decreased frequency of individuals with the low-expressing *MBL* genotype in Caucasian patients compared to control subjects. The same tendency was also observed in patients of other ethnic origin. The authors hypothesized that heterozygosity for *MBL2* variant alleles, responsible for low serum MBL levels, was associated with protection against clinical TB. Studies in Danish patients [13] and Turkish children [42] showed no association between *MBL2* polymorphisms at the codons 54 and susceptibility to TB. A large study in India showed that a significantly increased genotype frequency of *MBL* mutant homozygotes was seen in pulmonary TB patients as compared with that in control subjects [59].

Our results showed similar outcome as a study that carried out in PTB patients of Chines. In Wu et al. study, the *MBL* (*HH*) and (*HL*) genotype was associated with susceptibility to TB, although in our study *MBL* (*HL*) genotype were not associated with susceptibility to pulmonary tuberculosis [37].

In daCruz et al. study, the *BB* genotype frequency distribution was similar between patients and controls, but the *B* allele, as well as the *AB* genotype, was significantly more frequent in TB patients than healthy controls; the homozygous *AA* genotype, responsible for higher levels of circulating MBL proteins, was more frequent in controls than in TB patients [34]. In a Araújo et al. study, have studied the *AB MBL2* variant in TB patients and healthy controls in a population from the Amazon region (Northern of Brazil), without finding any evidence of association between this polymorphism and TB [60]. Shi et al. study reported for the MBL2 codon 54 *AB* gene polymorphism, significant association was found in the dominant model (OR = 1.52, 95% CI: 1.22–1.88), homozygote comparison (OR = 2.10, 95% CI: 1.08–4.09), and B vs. A (OR = 1.45, 95% CI: 1.20–1.75). The results suggested that individuals with *B* allele may have an increased risk of TB as compared with wild type *AA* homozygotes in Chinese population. In their study no significant association was found between *MBL2* + 4 *PQ* gene polymorphism and the risk of TB. They suggests that individuals carrying the *MBL2* codon 54 *B* allele may have an increased risk of TB as compared with *AA* homozygous, whereas *MBL2* + 4 *PQ* gene polymorphism is possibly not associated with TB in Chinese population [38].

TB is a multifactorial disease and its susceptibility depends not only upon host genetic aspects, but also on mycobacteria characteristics and on interactions with environmental factors. In addition, different genetic backgrounds and environmental factors may also influence the results. There is no doubt about the difficulty in comparing results from studies conducted in different populations, even when the same allele or haplotype are analyzed and the same study design is used. These paradoxical findings could be due to the Ethnic difference, different between genotyping technique, criteria for sample inclusion and exclusion and the small sample size of this study and could be caused by gene-environment interaction, gene-gene interaction, and gene-agent interactions [61], [62].

## 5 Conclusions

Our comprehensive analysis of *MBL* polymorphisms suggests that *MBL* (*HH*) genotype, *H* and *L* allele associate with PTB risk in the Lur population of Iran. For better understanding of the association between these polymorphisms and the risk of TB, larger sample size and more studies, especially those investigating haplotypes as well as gene-gene and gene-environment interactions, are required in the future. Further studies are required to determine how *MBL* polymorphisms influence susceptibility to Mycobacterium tuberculosis infection.

## Conflict of interest statement

The authors declare no conflict of interest.
